# Supplementary material for: Pleiotropy increases with gene age in six model multicellular eukaryotes
Source: bioRxiv. 2024 Nov 21:2024.11.19.624372. Preprint. [Version 1] doi: 10.1101/2024.11.19.624372 (PMC11601630; doi:10.1101/2024.11.19.624372)
Supplement: Supplement 1 — Table S1: ANOVA tables for the relationship between the number of protein-protein interactions a gene is associated with and the age of that gene. Table S2: Two-way ANOVA tables for the relationship between protein-protein interactions, gene age, and the primary trait associated with a gene. Table S3: Two-way ANOVA tables for the relationship between protein-protein interactions, gene age, and gene duplication. Figure S1: Old genes have an elevated prevalence of pleiotropy compared to young genes Figure S2: The prevalence of pleiotropy is dependent on the functions a gene participates in Figure S3: Genes with paralogs are more pleiotropic than genes without paralogs Figure S4: Bootstrapped mean biological process values for functional groups of genes Figure S5: Bootstrapped mean protein-protein interaction counts for functional groups of genes [file media-1.pdf]

Supplemental Material for *Pleiotropy increases with gene age in six model multicellular eukaryotes*

Authors: Martin, Reese[1,2*], and Tate, Ann.T.[1,2a]

[1]Department of Biological Sciences, Vanderbilt University, Nashville TN, 37235

[2]Evolutionary Studies Initiative, Vanderbilt University, Nashville, Tennessee, USA

*Email address: reese.a.martin@vanderbilt.edu

[a]Corresponding email address: a.tate@vanderbilt.edu

Table S1: ANOVA tables for the relationship between the number of protein-protein interactions a gene is associated with and the age of that gene.

Species	Formula	df	Sum. Sq.	Mean Sq.	F	PR(>F)
H. sapiens	PPI~Age	1	2.57e6	2.57e6	240.45	<2e-16
M. Musculus	PPI~Age	1	1.6e7	1.6e7	1218.54	<2e-16
D. rerio	PPI~Age	1	1.31e7	1.31e7	1291.64	<2e-16
D. melanogaster	PPI~Age	1	9.19e6	9.19e6	1422.42	<2e-16
C. elegans	PPI~Age	1	6.53e6	6.53e6	462.06	<2e-16
A. thaliana	PPI~Age	1	3.90e7	3.90e7	1678.95	<2e-16

Table S2: Two-way ANOVA tables for the relationship between protein-protein interactions, gene age, and the primary trait associated with a gene.

Species	Factor	df	Sum. Sq.	Mean Sq.	F	PR(>F)
H. sapiens	Age	1	647.40	647.40	540.59	<2e-16
	Traits	4	29.93	7.48	6.25	5.36e-5
M. Musculus	Age	1	1317.85	1317.85	1044.26	<2e-16
	Traits	4	51.53	12.88	10.21	3.30e-8
D. rerio	Age	1	726.31	726.31	600.13	<2e-16
	Traits	3	5.66	1.89	1.56	.19
D. melanogaster	Age	1	955.98	955.98	653.70	<2e-16
	Traits	3	39.71	13.24	9.05	5.82e-6
C. elegans	Age	1	1114.80	1114.80	591.81	<2e-16
	Traits	3	25.90	8.63	4.58	.0033
A. thaliana	Age	1	1770.34	1770.34	1303.98	<2e-16
	Traits	3	16.94	5.65	4.16	.006

Table S3: Two-way ANOVA tables for the relationship between protein-protein interactions, gene age, and gene duplication.

Species	Factor	df	Sum. Sq.	Mean Sq.	F	PR(>F)
H. sapiens	Age	1	2.83e6	2.83e6	268.22	<2e-16
	Duplication	1	1.16e6	1.16e6	109.65	<2e-16
M. Musculus	Age	1	1.58e7	1.58e7	1202.33	<2e-16
	Duplication	1	2.16e5	2.16e5	16.44	5.04e-5
D. rerio	Age	1	1.31e7	1.31e7	1290.98	<2e-16
	Duplication	1	6.33e4	6.33e4	6.23	0.013
D. melanogaster	Age	1	9.22e6	9.22e6	1428.41	<2e-16
	Duplication	1	1.09e4	1.09e4	1.68	0.19
C. elegans	Age	1	6.33e6	6.33e6	448.40	<2e-16
	Duplication	1	3.77e5	3.77e5	26.73	2.37e-7

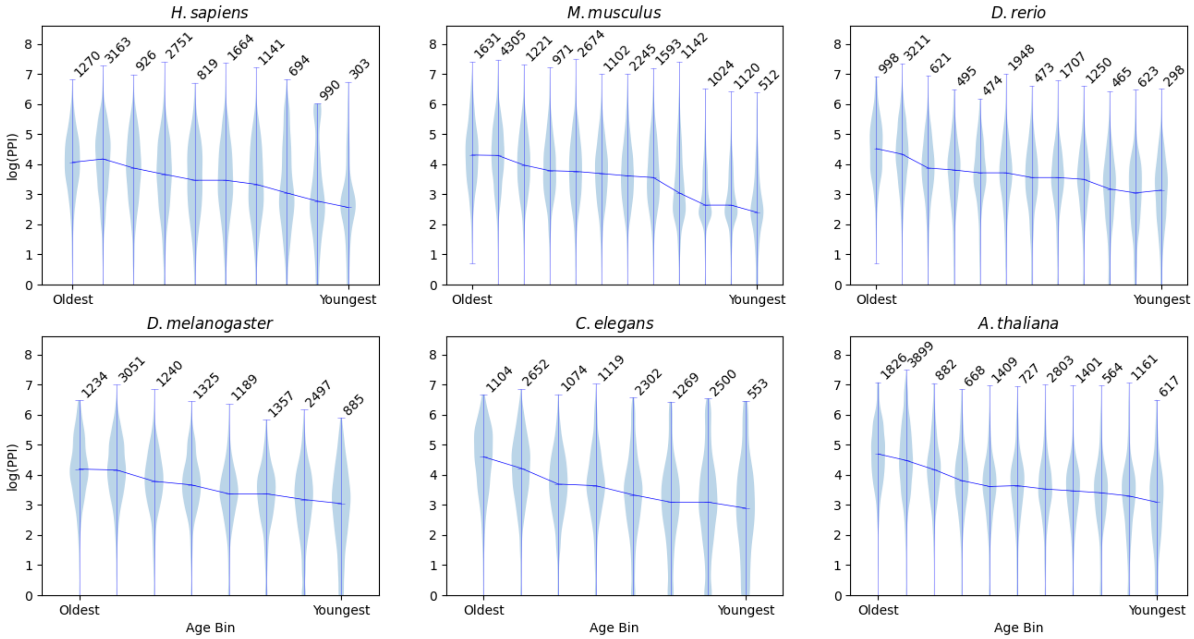

Figure S1: Old genes have an elevated prevalence of pleiotropy compared to young genes. The y axis shows violin plots built on the $\log_{10}$(PPI) for all genes in each age bin. The x axis shows age bins for genes, from oldest on the left to the youngest on the right. Orthologs that did not have a PPI count were excluded from these plots.

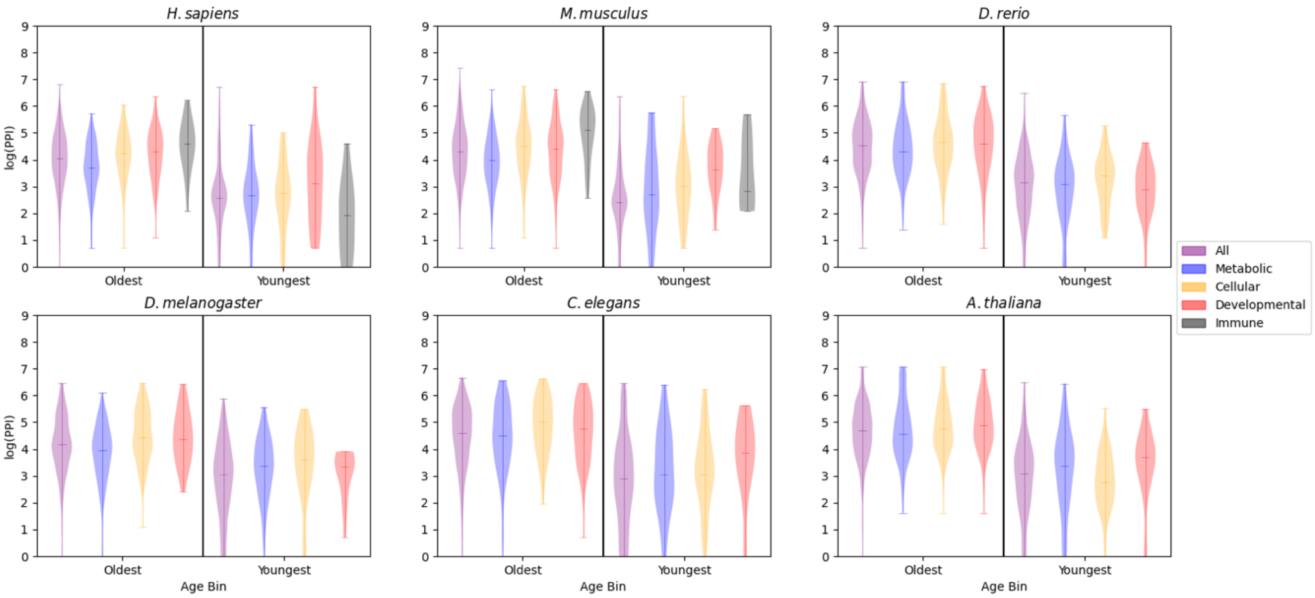

Figure S2: The prevalence of pleiotropy is dependent on the functions a gene participates in. Plots show genes in the oldest time bin and the youngest time bin (labeled Old and Young). The y axis shows violin plots built on the $\log_{10}$(PPI) for all genes in a given age bin, separated into 4 functional groups (metabolic, cellular process, developmental, and immune processes). Orthologs that did not have a PPI count were excluded from these plots.

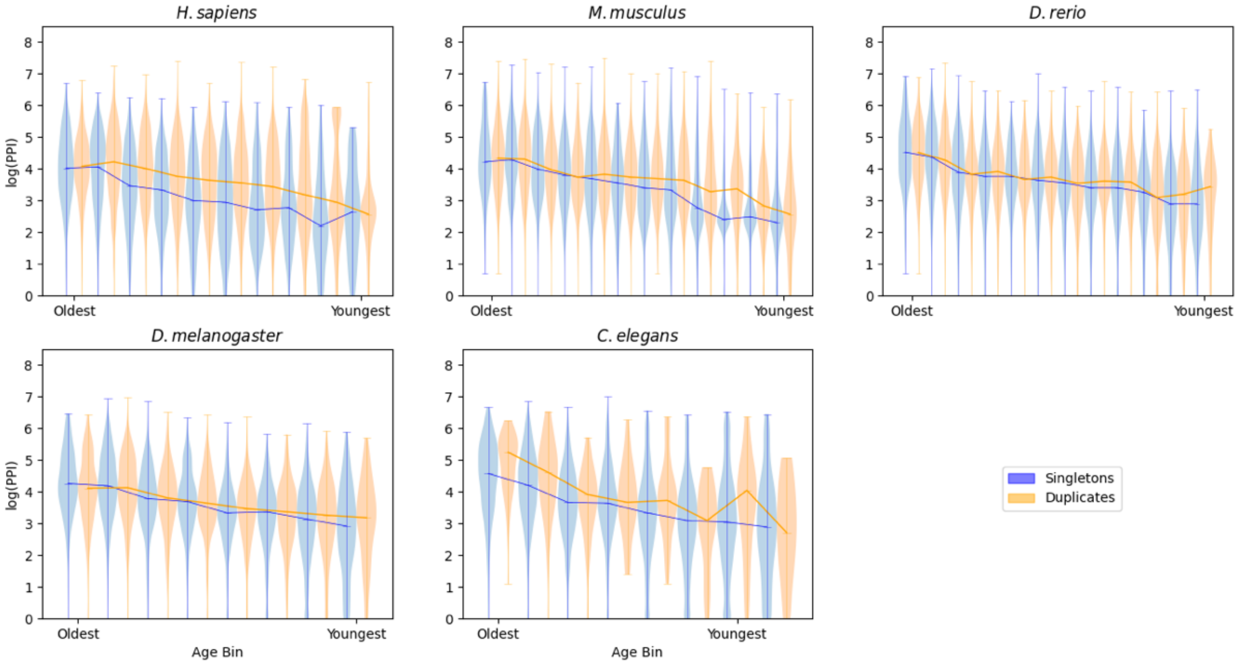

Figure S3: Genes with paralogs are more pleiotropic than genes without paralogs. The y axis shows violin plots built on the $\log_{10}$(PPI) for all genes in a given age bin. The x axis shows age bins for genes, from oldest on the left to the youngest on the right. Genes with paralogs are shown in orange, genes without paralogs are shown in blue. Orthologs that did not have a PPI count were excluded from these plots.

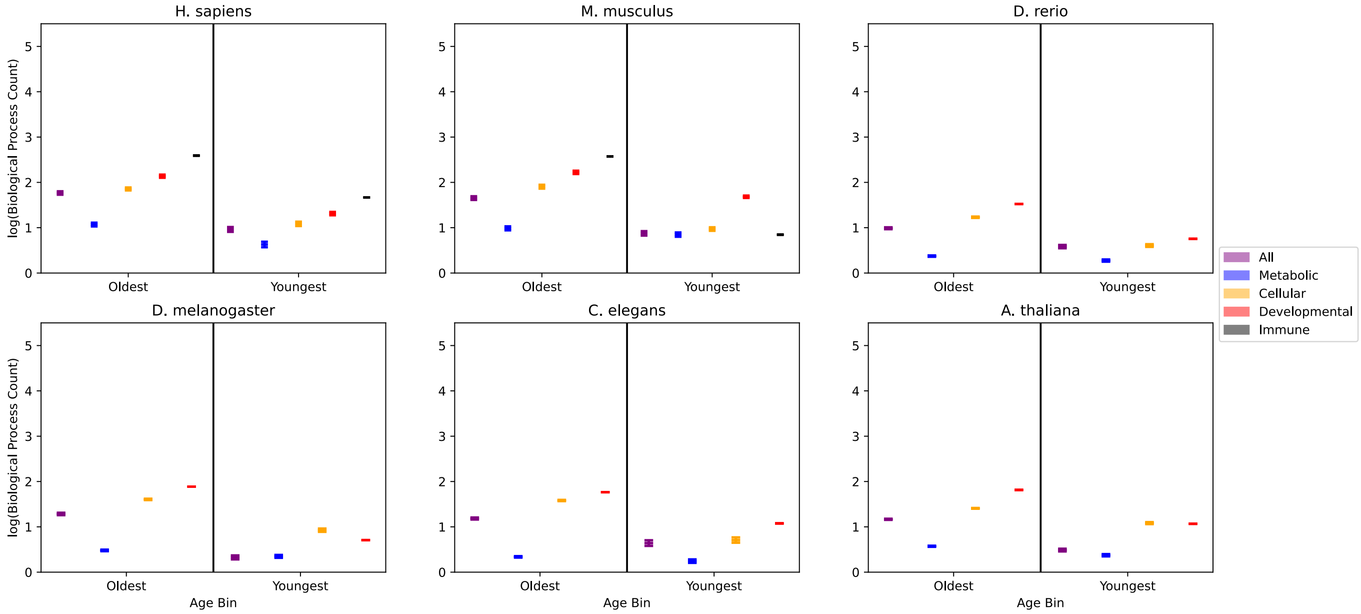

Figure S4: Bootstrapped mean biological process values for functional groups of genes

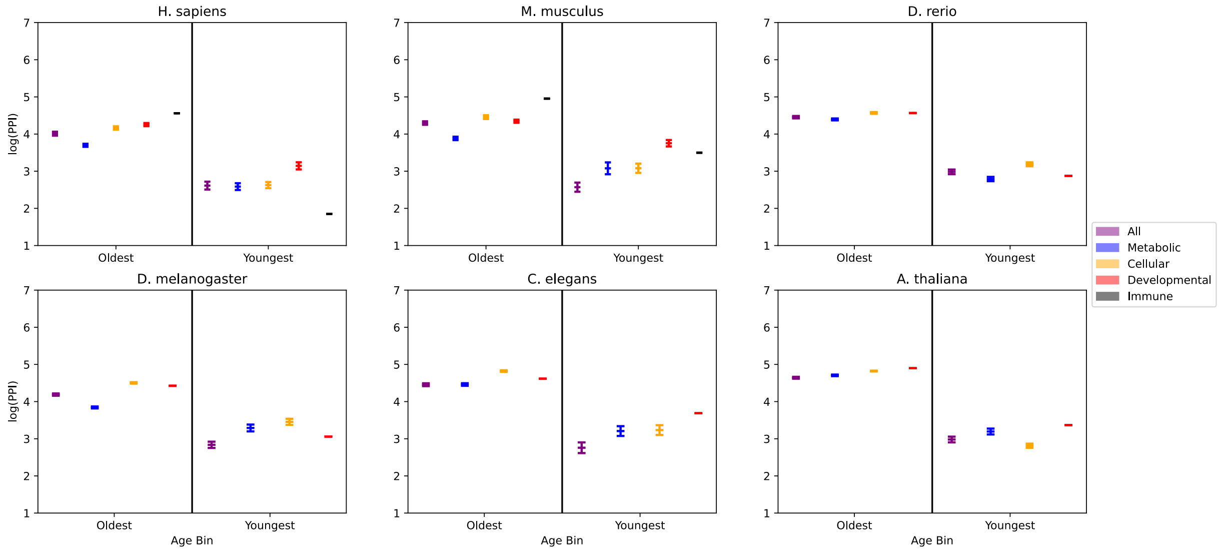

Figure S5: Bootstrapped mean protein-protein interaction values for functional groups of genes
